# Social Capital Promotes a Healthier Diet among Young Adults by Reducing Psychological Distress

**DOI:** 10.3390/nu14235187

**Published:** 2022-12-06

**Authors:** Brigita Mieziene, Arunas Emeljanovas, Dario Novak, Ichiro Kawachi

**Affiliations:** 1 Department of Physical and Social Education, Lithuanian Sports University, 44221 Kaunas, Lithuania; 2 The Faculty of Kinesiology, University of Zagreb, 10110 Zagreb, Croatia; 3 Department of Social and Behavioral Sciences, Harvard T.H. Chan School of Public Health, Boston, MA 02115, USA

**Keywords:** family support, social trust, social capital, health-related behavior

## Abstract

Studies have revealed the links between social capital and diet. However, the mediating role of psychological distress in this relationship has been understudied. This study aims to identify direct and indirect relationships between social capital and adherence to the Mediterranean diet among Lithuanian young adults and identify the mediating role of psychological distress in this relationship. Data were collected from 1336 young adults, aged 18–36 years; 40.5% were males. MEDAS was used to measure adherence to a healthy diet. Social capital was measured by eight separate items in terms of family support, social support, social cohesion, social trust, communication, collaboration, participation, and distant communication. Kessler’s six-item scale was used to assess psychological distress. Higher family support (β = 0.105), higher social participation (β = 0.294), and lower psychological distress (β = 0.073) directly predicted higher adherence to the Mediterranean diet. Social capital was indirectly related to adherence to the Mediterranean diet, with standardized effect sizes of 0.02–0.04, indicating small effect sizes. Thus, psychological distress mediates the relationship between social capital and a healthy diet. Given that social capital is related to psychological health and both directly and indirectly predicts healthy behavior in young adults, further longitudinal and experimental research is required to measure the effects of the intervention on incorporating, facilitating, encouraging, and implementing measures to strengthen the social connection between people and groups of people within the community, neighborhood, and organizations.

## 1. Introduction

A healthy diet is essential to maintain physical [1] and mental health [2]. Studies show that compliance with nutritional recommendations not only prevents chronic disease and premature mortality [1,3] but also helps to regulate immune homeostasis [4], which becomes particularly important during pandemics.

The Mediterranean diet has long been considered the gold standard of healthy nutrition, and it is recognized as a cultural heritage by the World Health Organization (WHO) and UNESCO [5,6]. In addition, the benefits of adherence to the Mediterranean diet for individual and public health are well studied and summarized in reviews [7] and meta-analyses [8]. As a result of globalization due to trade and travel and media effects, the Mediterranean diet could be implemented across the world and adopted by various populations.

The COVID-19 pandemic is a phenomenon that threatens global nutrition. Studies on dietary patterns during the COVID-19 pandemic showed an increased adherence to a healthy diet [9,10]. However, consumption of ‘unhealthy’ foods such as alcoholic drinks, snacks, candies, and commercial pastries increased as well [11]. People expressed an increased appetite during the pandemic, and as much as half of them gained weight [12].

Adherence to the Mediterranean diet varies depending on socioeconomic status, across age groups, and gender, with women having lower SES [13] and at younger ages [14]. Gender impact is inconsistent across studies, as some show better adherence in females, some in males, and others find no difference [15,16,17]. Aside from sociodemographics, which may help to adjust towards a healthy one, it is important to identify individual and interpersonal targets for interventions. 

Among individual psychological factors, psychological distress is a trigger for a poor diet. Emotions can control food choice, impair cognitive eating controls, and disturb impulse control in terms of food intake or food choice [18,19]. Craving for sweets and high-density foods is explained by their ability to improve mood and reduce stress [20]. Psychological distress is also positively related to takeaway food consumption frequency, while the desire for and consumption of foods lower in energy density (e.g., fruit and vegetables) decreases with distressed mood [21]. Findings suggest that, on average, participants’ depressive symptoms increased during COVID-19 [22]. Psychological distress in general, and COVID-19-related psychological distress in particular, can trigger a poor diet [23,24].

Meanwhile, interpersonal factors such as relationships with others, social support, trust, etc. might prevent or increase psychological distress [25]. Social capital summarizes these interpersonal aspects and can be defined as the sum of the resources of reciprocity, trust, and all kinds of support available to an individual within her social network [26]. Social capital is recognized as an important determinant of health-related behavior [27,28] through the beneficial effects of social support and knowledge obtained within the social environment [29]. Social capital becomes particularly important for an individual‘s health during times of crisis and uncertainty [30]. Since behavior can be modified by interpersonal factors, it is important to identify the target population-related social environment factors, as these factors might differ in different populations. The main distinction of young people of age 18 to 36 years old is that they are on the brink of (or recently separated from) their nuclear family and starting their own life along in a changing social environment. The social environment in adolescence that was often filled by family, classmates, and peers in the neighborhood is filtered out and changed or complemented by university friends, colleagues at work, life partners, and their families. Moreover, social capital is an integral part of the Mediterranean lifestyle. Having meals with friends and family and practicing conviviality is included in the latest version of the Mediterranean diet pyramid [31].

Studies before and during the COVID-19 pandemic revealed the links between social capital and diet. However, the mediating role of psychological distress in this relationship has not been clarified. The role of psychological distress as a mediator is supported by studies that showed the potential of social capital to reduce psychological distress [25] as well as studies that have linked psychological distress to diet [21,23].

Hence, this study sought to identify direct and indirect relationships between social capital and adherence to the Mediterranean diet among Lithuanian young adults and to identify the potential mediating role of psychological distress in this relationship.

## 2. Materials and Methods

### 2.1. Study Design and Procedure

Study participants in this cross-sectional study were enrolled using snowball sampling—a non-probability, convenience sample. Several researchers formed their initial samples from available participants (personal and professional contacts: university students, college students, members of youth organizations, representatives of professional societies, followers, and groups in social networks). The variety of participants was chosen to represent the various strata of the sample population according to the following criteria: gender, place of residence, family income, and education. Then, these participants were asked to enroll more participants in the study and share the survey’s internet link with their friends and colleagues—potential participants aged 18–36 years old. An online questionnaire was shared through popular social networks and emails within the period October 2020 - May 2021. The study procedure took approximately 15 min.

### 2.2. Participants

In total, data were collected from 1336 study participants, with 40.5% of them being men. The mean age was 22.31 (3.39), within the age ranging from 18-36 years. These and other sociodemographic characteristics of the study sample are described in the results (Table 1). Informed consent was provided along with the survey. All participants were informed about the goals of the study, the anonymity of their participation, and the option to cancel their participation at any time. Participants agreed to participate in the study by submitting their completed online survey questionnaire. The study was conducted following the Declaration of Helsinki, and the protocol was approved by the Lithuanian Sports University Ethics Committee (No. SMTEK-50).

### 2.3. Measurements

#### 2.3.1. Diet

The Mediterranean Diet Adherence Screener (MEDAS) [32] was employed to measure adherence to a healthy diet. The scale was previously validated in adult populations in other Mediterranean and non-Mediterranean counties [32,33] and used to measure dietary patterns in the Lithuanian sample of young adults in the previous study [34]. Two out of 14 items represent dietary habits: the use of olive oil and the preference for white meat instead of red meat. The remaining 12 items cover the amount of consumption of both healthy (olive oil, vegetables and fruits, fish, nuts, and dishes with homemade sauce) and unhealthy (animal fat, commercial pastries, sugar-sweetened beverages) food items. Each item was scored 0 (does not meet the healthy eating criteria) or 1 (meets the healthy eating criteria), following the thresholds for the health-related consumption of specific food items [35]. The total score was calculated by summing all item scores. The total score on the MEDAS scale indicates three categories of dietary patterns: ≤7 indicates low adherence, 8–9 indicates medium adherence, and ≥10 indicates high adherence to the Mediterranean diet [32].

#### 2.3.2. Social Capital

Each of the eight social capital indicators was measured by a separate item [36,37,38]. For bonding social capital identification, participants were asked to agree with the statement on a Likert scale from 1—“Not at all” to 5—“Very much”. Family support was evaluated by providing the statements, “Your family cares and understands you”, social support- “People in your environment help each other”, social cohesion—“People around you get along well with each other”, and social trust—“You can trust your friends”. Social interaction was measured by the frequency of social contact with other people in terms of communication (how often do you chat with others?), collaboration (how often do you interact working together with other people?), participation (how often do you participate in social activities with others?), and distant communication (how often do you communicate through telephone or internet?) with answers from 1—“Never” to 5—“Every day”.

#### 2.3.3. Psychological Distress

Kessler’s six-item scale was used to assess psychological distress [39]. On a scale from 0 (none of the time) to 4 (all the time), participants evaluated their nervousness, hopelessness, anxiety, restlessness or fidgety feelings, worthlessness, and depression. The total score was obtained by summing the scores for each item. A lower score indicates a lower level of psychological distress. The internal consistency of the scale was good (Cronbach α = 0.904). For the descriptive statistics, the summed score was dichotomized as low psychological distress (0–12 points) and high psychological distress (≥13 points) [39].

#### 2.3.4. Covariates

Participants had to indicate their full age for age evaluation. By designating themselves as living in a city or a region, participants indicated their place of residence. Financial status was evaluated by participants, who designated their household income as lower, the same, or higher than average compared with other households in their country. The current main occupation was identified by pointing out participants’ current positions as student, working, unemployed, or other. Afterward, as most of the study participants were students, occupation was binarized into students (category 1) and others (category 2). Education was evaluated by asking respondents to indicate the highest achieved degree at the moment of the survey with the answers 1—“High school”, 2—“Vocational”, 3—“Higher non-university degree”, and 4—“Higher university degree”. Family status was evaluated by participants, who indicated if they were single or had a life partner. Similarly, participants, by indicating if they live alone, with a spouse/partner, with parents, or with roommates, pointed out their cohabitation status.

### 2.4. Statistical Analysis

Data were analyzed using SPSS 28.0 software (SPSS Inc., Chicago, IL, USA). Descriptive statistics for determining the means, standard deviations, and frequency distributions of variables were used in the study. The chi-square test was employed to identify relationships between nominal and categorical study variables. The prediction of adherence to the Mediterranean diet was identified using hierarchical linear regression analysis. The skewness and kurtosis of the standardized residuals in the regression analysis were in the range between −1 and 1. PROCESS version 3.5. [40] SPSS macro (Model 4) was employed for mediation analysis. The direct effect of the mediator and indirect effect of the predictor on the outcome variable were estimated, along with their 95% confidence intervals (CI). An effect was considered significant when the CI did not include zero. Bootstrapping was set at 5000 samples. The completely standardized indirect effects were calculated as effect sizes for mediation [41]. Their values of 0.01, 0.09, and 0.25 represent small, medium, and large effect sizes, respectively [42]. Statistical significance was set at a *p*-value of less than 0.05.

STROBE Statement: checklist guidelines were followed in organizing this paper.

## 3. Results

The characteristics of the study sample are summarized in Table 1. Roughly half of the participants were in a relationship with a spouse or partner. Almost seven out of ten were students, a quarter worked, and the rest were unemployed or other. Somewhat more than half were secondary school graduates; a quarter of participants had higher education; around 14% graduated college; and about 7% had vocational education. Around 60% of participants rated their financial status as average, while one out of ten indicated lower-than-average financial status. Almost one-third of study participants lived with their parents, another 30% with a spouse or partner, one out of five were living alone, and around 15% were living with roommates. Almost half of the young adults perceived high psychological distress. As many as 60% of study participants reported low adherence to the Mediterranean diet, while only around 2% reported high adherence. Among social capital indicators, the most highly evaluated were family support communication and social trust.

Correlation analysis revealed that the Mediterranean diet, psychological distress, and social capital are all positively interrelated. The higher the rating of social capital in each domain, the higher adherence to the Mediterranean diet, and the lower the psychological distress among young adults. The strongest correlations are observed between higher adherence to the Mediterranean diet and higher social participation, as well as between lower psychological distress and participation (Table 2).

In the adjusted regression models (Model 1, Table 3), it is revealed that females, those having a spouse or partner, students, young adults being educated at a higher-level institution, and those having higher financial status, had better adherence to the Mediterranean diet. Female gender and higher financial status remained significant in other models (Models 2 and 3, Table 3). Only family support and social participation predicted higher adherence to the Mediterranean diet (Model 2, Table 3). Both social capital variables remained statistically significantly associated with the Mediterranean diet after the inclusion of psychological distress as a covariate. Lower psychological distress also predicted higher adherence to the Mediterranean diet among young adults (Model 3, Table 3).

Finally, an analysis of the mediation effect of psychological distress between social capital components and adherence to the Mediterranean diet was performed (Table 4). Each social capital variable was significantly related to psychological distress (a direct effect). The direct standardized effect between psychological distress and the Mediterranean diet was β = 0.17. Therefore, the criteria for mediation, that the predictor variable should be significantly related to the mediator and the mediator should be significantly related to the outcome variable, were satisfied in all mediation models. Family support, social support, social cohesion, social trust, communication, collaboration, participation, and distant communication were indirectly related to adherence to the Mediterranean diet, as all CIs for its βs do not cross 0. Standardized effect sizes were between 0.02 and 0.04, indicating small effect sizes.

## 4. Discussion

This study aimed to investigate the direct relationships between social capital and adherence to the Mediterranean diet as well as the mediating effect of psychological distress in this relationship.

Mental health has been shown to be important for maintaining healthy behavior in other studies [43]. Hence, deterioration of mental health can trigger a cascade of unhealthy behavioral changes, including more smoking and drinking as well as a decline in dietary quality. The COVID-19 pandemic brought about a significant challenge for different populations around the world. Both mental and physical health deteriorations were observed across countries [22,27,44]. The current study also found that 44% of young Lithuanian adults experienced high psychological distress, which is twice as high as pre-pandemic numbers among high school students in Lithuania [45]. Meanwhile, lower psychological distress was associated with better adherence to the Mediterranean diet during the COVID-19 pandemic confinement. The link is important to consider for health-behavior intervention programs, given that the average adherence to the Mediterranean diet among young adults was so low (only 2% reported being adherent). Other pre-pandemic studies also reported that higher levels of distress are associated with less frequent consumption of fish, vegetables, and fruit [46]. These results are complemented and confirmed by recent studies where higher adherence to the Mediterranean diet was linked to a lower intake of unhealthy food items stemming from higher psychological distress [47,48]. According to the authors, stress-related cortisol and insulin release in the body may stimulate ingestion of energy-dense “comfort foods”, thus improving mood and mitigating effects of stress-induced dysfunction and associated depression via brain opioidergic and dopaminergic neurotransmission [20].

Anyway, the relationship between mood and food choice in scientific literature is presented as bidirectional. Among explanations of the link between mental health and diet, the negative impact of distress on a diet is because of the reduced amount and frequency of food, which is said to be the ‘‘natural’’ response to stress and intense emotional states through both psychological and physiological mechanisms [46]. There is also a reverse explanation of the relationship between diet and general mental health, given that diet is increasingly implicated as a predictor of mental health. For example, depression and psychological distress are related to increased inflammatory processes in the body [49]. Moreover, those inflammatory processes are induced by increased consumption of sweet foods under stress and also in those who eat less overall while under stress [20].

Also, consuming red meat and drinking sugary drinks daily above recommended amounts and consuming nuts and vegetables below recommended amounts are related to higher depressive symptomatology [50].

Maintaining mental health becomes particularly important during times of crises such as the COVID-19 pandemic, but not exclusively, as populations around the world face many challenges locally or globally. Interpersonal factors have been important predictors of both mental health and health behavior [25,51] and during local or global crises [30]. The fact is confirmed by the current study, which found that higher levels of bonding social capital (family and social support), trust, and social cohesion, as well as social interactions (communication, collaboration, and social participation), were correlated with lower psychological distress and better adherence to the Mediterranean diet. Family support and social participation are important direct predictors of the diet even when psychological distress and socio-demographic covariates were considered. The direct relationship between family support and diet might be explained by shared values within the family and the care obtained from other family members. Although it was measured as general support (various forms of aid individuals receive or perceive from their family members, including emotional support, instrumental support, knowledge, and skills) and did not specifically address healthy nutrition per se, nevertheless, the effect of family support might affect diverse areas of daily life [28]. Moreover, given the pandemic-related restrictions and limited social contacts, the family became the most important source of access to social capital due to its proximity. The direct association between social participation and adherence to the Mediterranean diet has support in other observational research studies, which state that social participation can influence people to engage in healthy and prevent unhealthy behavior by providing knowledge and new skills for healthy behavior in a social environment [52].

Besides family support and social participation, other bonding and social interaction indicators were indirectly positively associated with adherence to a Mediterranean diet, mediated by psychological distress. Other studies also found that the effect of social capital on health-related outcomes was mediated by mental health factors such as stress and anxiety [53]. This chain of relationships is important to acknowledge because it points to the importance of the social environment as a determinant of mental health and health behavior, especially during times of crisis. Social interaction provides access to the resources that being with and communicating with others could provide. Bonding social capital influences mental health through access to support; social interaction provides belongingness and life meaning under the condition of positive social relationships. Contrary to this, an online study carried out among Colombian adults found that low social capital was associated with depression risk, elevated suicide risk, high perceived stress related to coronavirus disease, and insomnia risk [54]. In crises other than the COVID-19 pandemic, social capital protects mental health as well. For instance, after the Great East Japan Earthquake of 2011, for people who had even severe disaster damage, high social capital played an important role in protecting mental health [55].

There are several pathways by which social capital is linked to health. On a macro level, it influences health policy decision-making, and on a micro level, it provides health-related information and access to health resources. Among these and other pathways that social capital links to health, there are those related to mental health, such as reinforcing psychological resources such as self-esteem, supplying emotional support, and decreasing exposure to stressors [56]. By contrast, low social capital could trigger feelings of insecurity, helplessness, unpredictability, and uncertainty [53]. In the context of health-related predictors or outcomes, social capital should always be considered a target of interventions as it continues to prove to be an important direct or indirect predictor of health-related outcomes. Moreover, social capital has complex effects as it interferes with mental and physical health.

Among socio-demographic covariates in the current study, only gender and financial status remained significantly associated with the Mediterranean diet after controlling for social capital and psychological distress. In previous studies, adults adhering to a Mediterranean diet were more likely to be older and have a higher educational status [10,57,58]. In our study, students had better adherence than non-students. This might be explained firstly by our sample (predominantly females and students), and secondly, even though they have not yet graduated, students are on their way to receiving higher education. Moreover, the students in our sample who were enrolled in public health and sports studies might receive the benefit of nutritional education. People in partnerships also have better adherence to healthier diets [46]. Partnership or marriage usually includes sharing meals. Other studies found that sharing meals in young adulthood is associated with a greater intake of fruit, vegetables, milk products, and some key nutrients [59]. Being in a partnership induces the mutual reinforcement of healthy behavior. This emphasizes the importance of interventions that target dyads, suggesting that the adoption of exercise or diet modifications by one individual is likely to spread to the other [60].

Gender links to adherence to a healthy diet are a controversial topic, as some studies report higher adherence among men [58], while other studies report the opposite [14,61]. Meanwhile, some studies report no significant differences between women and men [62]. Higher financial status was a predictor of a better diet in this and other studies [63,64]. High adherence to the Mediterranean diet is typically associated with higher costs, spent on vegetables, fruits, nuts, fish and seafood, and olive oil, which are usually more expensive than junk food or other traditional items in the Western diet. However, researchers found that greater adherence to the Mediterranean diet is associated with only slightly higher dietary costs than the Western-type diet, and this might be explained by the reduction of expenses spent on unhealthy food [13].

### Limitations

As the study is cross-sectional, the direction of links between phenomena is based on theoretical premises and common sense. The precise direct and indirect effects of social capital on diet could be measured in experimental and longitudinal studies. The results are mostly represented by students, as they dominated among study participants. Snowball convenience sampling instead of representative random sampling was used. Thus, some groups of respondents might be underrepresented. However, due to the COVID-19 restrictions at the time of data collection and difficulties reaching the target population, which is the case in this study, snowball sampling is recommended as an option [65]. Finally, the study did not represent the detailed diet of Lithuanian young adults, as the dietary tool used was a diet screener and could provide an in-depth dietary assessment.

## 5. Conclusions

Social capital increases the probability of better adherence to a healthy diet by reducing psychological distress among young adults, predominantly students. As social capital encourages healthy nutrition in times of global crisis, it becomes particularly important for mental and physical health to build and maintain social capital. In addition, group-based healthy nutrition programs should be initiated in universities and other organizations to provide young employees with the opportunity to build or maintain social capital, adapt to social norms, and improve their knowledge and skills in healthy nutrition. Bonds strengthened through social interaction may buffer psychological distress, thereby keeping young adults more adherent to healthy nutrition. Further longitudinal and experimental research is required to measure the effects of the intervention on incorporating, facilitating, encouraging, and implementing measures to strengthen social connections between people and groups of people within the community, neighborhood, and organizations.

## Figures and Tables

**Table 1:** Descriptive statistics of study variables.

Study Variables	% or Mean (SD)
Sex	
Men	40.5
Women	59.5
Age	22.31(3.39)
Family status	
Single	43.3
Have a spouse/partner	56.7
Main occupation	
Student	69.2
Employed	25.5
Unemployed	3.8
Other	1.5
Education	
Secondary	54.0
Vocational	6.9
College	14.2
Higher	24.9
Financial status	
Lower than average	11.3
Average	58.5
Higher than average	30.1
Cohabitation	
Alone	22.1
With a spouse/partner	30.2
With parents	32.6
With roommates	15.1
Psychological distress	
Total score (points range 0–24)	14.31 (5.48)
Low	55.8
High	44.2
Mediterranean diet	
Total score (points range 0–14)	5.12 (2.02)
Low	60.4
Medium	37.7
High	1.9
Social capital	
Family support (points range 1–5)	4.32 (0.10)
Social support (points range 1–5)	3.91 (0.96)
Social cohesion (points range 1–5)	3.93 (0.87)
Social trust (points range 1–5)	4.13 (0.79)
Communication (points range 1–5)	4.24 (0.91)
Collaboration (points range 1–5)	3.74 (0.10)
Participation (points range 1–5)	3.77 (0.97)
Distant communication (points range 1–5)	4.19 (0.95)

**Table 2:** Correlations between Mediterranean diet, psychological distress, and social capital in young adults.

	Family Support	Social Support	Social Cohesion	Social Trust	Communication	Collaboration	Participation	Distant Communication	MD Total
MD total	0.228 **	0.198 **	0.193 **	0.191 **	0.145 **	0.214 **	0.324 **	0.097 **	1
PD	0.189 **	0.201 **	0.211 **	0.205 **	0.159 **	0.140 **	0.272 **	0.076 *	0.192 **

Note: MD—Mediterranean diet; PD—psychological distress; * *p* < 0.05; ** *p* < 0.01.

**Table 3:** Prediction of adherence to the Mediterranean diet from sociodemographic, social capital, and psychological distress factors.

Variable	Model 1	Model 2	Model 3
Sex (female)	0.094 **	0.123 ***	0.133 ***
Age	0.053	0.081 *	0.067
Family status (has a partner/spouse)	0.070 *	0.048	0.051
Occupation (other than student)	−0.109 **	−0.032	−0.033
Education	0.093 *	0.056	0.056
Financial status	0.185 ***	0.117 ***	0.110 ***
Cohabitation (not alone)	−0.030	−0.035	−0.031
Family support		0.105 **	0.097 **
Social support		0.018	0.016
Social cohesion		−0.021	−0.023
Social trust		0.044	0.039
Communication		−0.010	−0.015
Collaboration		0.047	0.045
Participation		0.294 ***	0.282 ***
Distant communication		−0.044	−0.046
Psychological distress			0.073 *
ΔR^2^; *p*	0.07; <0.001	0.12; <0.001	0.01; 0.028

Note: * *p* < 0.05; ** *p* < 0.01; *** *p* < 0.001; Model 1: socio-demographic covariates; Model 2: social capital predictors adjusting for socio-demographic variables; Model 3: psychological distress as a predictor adjusting for socio-demographic and social capital variables.

**Table 4:** Direct and indirect effects of social capital indicators on psychological distress (direct) and adherence to the Mediterranean diet (indirect).

Pathways from:	Effect	Pathway to PD		Pathway to MD via PD	
		β	95% CI	β	95% CI
Family support	Direct effect	0.55	[0.32–0.78] ***	0.13	[0.05–0.22]
	Indirect effect			0.04	[0.02–0.06]
	CSIE			0.02	[0.01–0.03]
Social support	Direct effect	1.20	[0.92–1.48] ***	0.22	[0.11–0.33]
	Indirect effect			0.07	[0.04–0.11]
	CSIE			0.03	[0.02–0.05]
Social cohesion	Direct effect	1.23	[0.92–1.54] ***	0.28	[0.16–0.40]
	Indirect effect			0.07	[0.04–0.11]
	CSIE			0.03	[0.02–0.05]
Social trust	Direct effect	1.53	[1.19–1.89] ***	0.31	[0.18–0.44]
	Indirect effect			0.09	[0.05–0.13]
	CSIE			0.04	[0.02–0.05]
Communication	Direct effect	1.10	[0.80–1.40] ***	0.19	[0.07–0.31]
	Indirect effect			0.07	[0.04–0.10]
	CSIE			0.03	[0.02–0.04]
Distant communication	Direct effect	0.64	[0.35–0.93] **	0.14	[0.03–0.25]
	Indirect effect			0.04	[0.02–0.07]
	CSIE			0.02	[0.01–0.03]
Collaboration	Direct effect	0.77	[0.49–1.05] *	0.34	[0.24–0.45]
	Indirect effect			0.05	[0.02–0.07]
	CSIE			0.02	[0.01–0.03]
Participation	Direct effect	1.42	[1.15–1.69] ***	0.47	[0.36–0.57]
	Indirect effect			0.07	[0.04–0.10]
	CSIE			0.03	[0.02–0.05]

Note: * *p* < 0.05; ** *p* < 0.01; *** *p* < 0.001, PD—psychological distress; MD—Mediterranean diet; CI—confidence interval; CSIE—completely standardized indirect effect.

## Data Availability

Not applicable.
